# Identification and Repair of Left-Sided Paraduodenal Hernia Using Both Laparoscopic and Robotic Techniques

**DOI:** 10.1155/2020/7569530

**Published:** 2020-02-05

**Authors:** Muhonen John, Hsu Michael, Sturdivant Matthew, Unger Anthony, Dexter David, Giuseppucci Pablo, Esper Christopher

**Affiliations:** ^1^UPMC Horizon, Farrell, PA, USA; ^2^LECOM, Erie, PA, USA; ^3^UPMC Hamot, Erie, PA, USA

## Abstract

Internal hernias are an uncommon cause of small bowel obstruction and present a challenging clinical diagnostic scenario. They result from the abnormal protrusion of an abdominal organ through a peritoneal defect and can cause intermittent obstructive symptoms, diffuse abdominal discomfort, and postprandial pain. Paraduodenal hernias comprise 53% of all internal hernias ^1^ and occur due to failure of the fixation of either the left or transverse mesocolon to the posterior abdominal wall. Its relative rarity results in mortality between 20 and 50% ^2^ because of delayed diagnosis and consequent obstruction, strangulation, and bowel ischemia. Our case series describes three patients before and after operative fixation of paraduodenal hernia. Only one of the three was identified by preoperative radiologist interpretation. Subsequent diagnosis and definitive treatment were completed by surgical staff to resolve undiagnosed undulating abdominal pain and obstructive-type symptoms. We further analyze left-sided paraduodenal hernias after laparoscopic and robotic repair to define common symptomatology, typical CT findings, and preferred laparoscopic repair techniques.

## 1. Introduction

**Figure 1:**
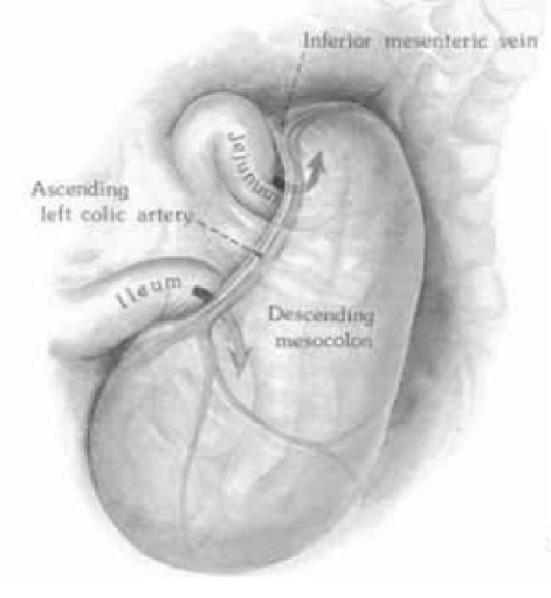
Left PDH: small bowel herniation into the fossa of Landzert formed from the incomplete fusion between mesentery and parietal peritoneum [22].

**Figure 2:**
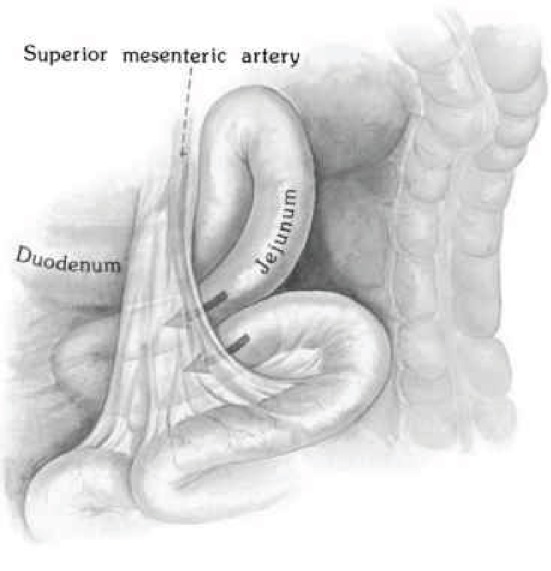
Right PDH: small bowel herniation into Waldeyer's fossa, a transverse colonic defect formed from proximal midgut nonrotation [22].

Internal hernias are a rare cause of small bowel obstruction that result from the abnormal protrusion of an abdominal organ through a peritoneal defect. Paraduodenal hernias (PDH) arise as a result of the failure of fixation of either the left or transverse mesocolon to the posterior abdominal wall and account for 53% of all internal hernias [1]. Although uncommon, paraduodenal hernias carry a mortality rate between 20% and 50% [2] as a result of delayed diagnosis and associated volvulus, ischemia, strangulation, and obstruction [3]. Paraduodenal hernias have been discovered in 0.2 to 0.9% of the population [4] on autopsy, and left-sided PDH account for 75% of all paraduodenal hernias [5]. They result from the dorsal malrotation of the midgut, an incompletely fixed left mesocolon and subsequent failure of peritoneal fusion. This produces a hernia which protrudes into the fossa of Landzert, an unusual congenital peritoneal fossa found behind the descending mesocolon and bordered anteromedially by the inferior mesenteric artery and vein, and the ascending branch of the left colic artery (Figure 1) [6]. Normal cecal and colon anatomic position is maintained with left-sided PDH. A right-sided paraduodenal hernia is formed from proximal midgut nonrotation. Normal anatomy is disturbed, and the cecum remains adhered in the right upper quadrant by Ladd's bands. The small bowel herniates through the transverse colonic mesenteric defect into Waldeyer's fossa, and the resulting hernia sac contains the ileocolic, right colic, and middle colic vessels within the anterior wall and the superior mesenteric artery and vein along its medial border (Figure 2) [2]. Waldeyer's fossa is located inferior to the 3rd portion of the duodenum, behind the small bowel mesentery, and its orientation to the right inferior lateral region displaces the right colic vein [7].

Symptoms of PDH are often nonspecific, intermittent, and encompass many acute and chronic obstructive-type abdominal complaints. Patients can be entirely asymptomatic or describe symptoms ranging from intermittent abdominal pain due to spontaneous hernia reduction [8] to an acute abdomen with associated strangulation and bowel necrosis. Only 50% of patients recall previous nonspecific recurrent abdominal pain [9], typically worse after meals and improved with body position [5]. There will be no specific exam or laboratory findings to differentiate PDH from other pathology [10] beyond elevated inflammatory markers typically associated with worsening bowel ischemia. Mean age at PDH diagnosis is 38.5 years [2], and males are affected three times more frequently than females [9]. The vague presentation of this type of internal hernia requires a high index of suspicion by the surgical team due to poor clinical outcomes from delayed or missed diagnosis [11].

Computed tomography (CT) is the gold standard for diagnosis for PDH [12] but only correctly identifies pathology in 43% of cases [13]. PDH will appear on CT as a cluster of dilated bowel segments, variably with an intestinal closed loop and associated engorged and displaced mesenteric vessels around the hernia orifice and sac [14]. Understanding the anatomy associated with left and right PDH is paramount. Subtle findings such as positioning of mesenteric fat and vessels as well as regional associations with other vessels can alter diagnosis and treatment [15]. The use of other imaging modalities is described in the literature. Upper GI studies will show dilated, herniated loops in one anatomic position with delayed contrast emptying but will often be nonspecific [16]. Ultrasound may show features of internal hernia; however, it is highly operator dependent and requires subsequent definitive imaging. Plain films are nonspecific albeit the initial imaging technique. They will typically show dilated loops of small bowel as well as air fluid levels, typical of a small bowel obstruction without indication of etiology [17].

Time from onset of initial symptoms to diagnosis does not differ significantly between right- and left-sided PDH [1]. Definitive treatment involves laparoscopy or exploratory laparotomy for direct visualization, reduction, possible lysis of adhesions to release of bowel from the hernia sac, reorientation of small bowel segments to their normal anatomical positions, repair of the hernia defect with suture or widening to avoid future obstruction, and, rarely, application of mesh [18]. Due to developmental differences, left PDH are amenable to uncomplicated manual reduction, whereas right PDH are more frequently complicated by strangulation. These require surgical release of the hernia sac due to closely associated Ladd's bands and possible right-sided medial visceral rotation [19]. Repair of the hernia defect between the colonic mesentery and the parietal peritoneum versus widening is subject to debate, although most literature endorses primary repair of defect.

## 2. Radiographic Identification

**Figure 3:**
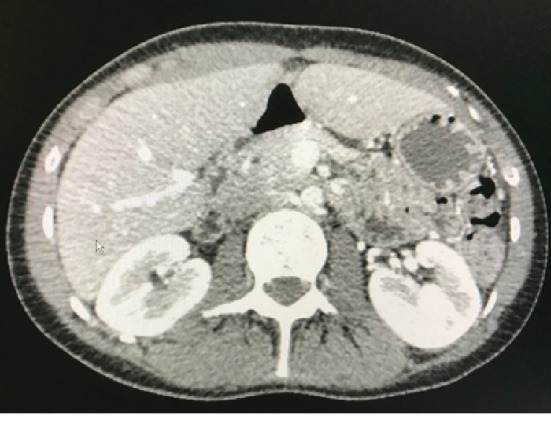
Axial view, left paraduodenal hernia.

Left paraduodenal hernias occur more frequently than right paraduodenal hernias. They result from herniation of the small bowel behind the forth portion of the duodenum into the fossa of Landzert due to incomplete fusion between inferior mesentery to the parietal peritoneum [20]. There are several distinctive features on CT scan to identify left PDH. The most obvious abnormality is the displacement and collection of the small bowel to the left upper quadrant lateral to the duodenum. The resultant mass effect causes anterior displacement of the posterior stomach and inferior migration of the transverse colon and duodenojejunal flexure (Figure 3) [21]. Vascular abnormalities include the anterolateral displacement of the inferior mesenteric vein and ascending left colic artery and generalized engorgement, grouping, and stretching of the main mesenteric trunks [21].

Right paraduodenal hernias occur with small bowel herniation through the Waldeyer's fossa. Although infrequent, they are more likely to occur in setting of nonrotated small bowel or malrotated right colon [20]. Similar to left PDH, CT scan of right PDH will show a saclike mass of small bowel located posterior to the superior mesenteric artery and inferior to the third part of the duodenum [20]. There is characteristic left lateral displacement of surrounding structures and inferior migration of the ascending colon. Deviation of normal anatomy is proportional to the extent of malrotation. Characteristically, the SMA and right colic vein will be found in the anteromedial portion of the herniated small bowel and the SMV will be found anterior left lateral to the superior mesenteric artery with loss of horizontal orientation of the duodenum [20].

## 3. Case Reports

### 3.1. Case 1

A 29-year-old male patient presents with 24 hours of postprandial mid and left upper quadrant abdominal pain. The patient endorsed nausea without emesis, prior laparoscopic appendectomy 2 years ago, and concerns for recurrent small bowel obstruction previously evaluated on multiple occasions. During prior workup, CT imaging showed concerns for partial small bowel obstruction; however, repeat studies after hospitalization showed free flow of contrast and subsequent symptom relief. There was no suggestion of internal hernia per official radiology reports. Patient lab work on presentation was unremarkable, and repeat CT scan showed a large amount of small bowel in the left upper quadrant with swirling of the mesentery and retention of oral contrast. The patient was diagnosed with internal hernia and taken to the OR for diagnostic laparoscopy. During evaluation, the small bowel was run from the cecum to the ligament of Treitz and a 45 cm segment of the small bowel was found to reside behind the transverse mesocolon. After reduction, the patient was noted to have a 4 cm defect along the left lateral side of the fourth portion of the duodenum just distal to the ligament of Treitz. The defect was repaired with an Endo Stitch in typical fashion, and the patient was discharged on postoperative day #3 following an unremarkable postoperative course. Follow-up over the next 18 months showed complete relief of previous recurrent intermittent abdominal pain and no suggestion on repeat imaging for return of the paraduodenal hernia.

### 3.2. Case 2

A 39-year-old female patient with a past medical history only notable for GERD, anxiety, and tubal ligation presented to the emergency department multiple times over the course of 5 days for worsening epigastric abdominal pain with associated nausea and gastric emesis. The patient admitted to radiation of her pain to the left upper abdomen and worsened symptoms with meals. The patient endorsed a history of chronic epigastric and left upper quadrant abdominal pain that has been going on for many years, was evaluated multiple times, and felt to be related to GERD. CT abdomen and pelvis with IV and oral contrast during the last ER visit showed the small bowel contained in the lesser sac with anterior displacement of the lesser curvature of the stomach without obstructive-type appearance on imaging. According to the radiologist, it was an unremarkable scan. The diagnosis of left PDH was made following surgical evaluation and secondary review of imaging. The patient was subsequently taken to the operating room for diagnostic laparoscopy and was found to have a 3-4 cm defect and approximately 30 cm of small bowel behind the mesentery of the transverse colon. This was subsequently reduced, and the Endo Stitch was used to repair the defect in typical fashion. The patient had a benign follow-up visit 1 month after the procedure and in the 9 months since has been entirely asymptomatic.

### 3.3. Case 3

A 29-year-old female patient presented to the acute care service with 1 day of periumbilical and right lower quadrant abdominal pain and complaints of nausea, gastric emesis, and nonbloody loose stools. Labs upon initial evaluation were unremarkable, and radiologist review of CT scan of the abdomen and pelvis was notable for a nonvisualized appendix without concerning signs for obstruction or internal hernia. Transvaginal ultrasound was unremarkable. It was felt that the patient's thin nature made CT diagnosis of appendicitis difficult. Due to the patient's extreme tenderness on physical exam, she was taken to the operating room for a laparoscopic appendectomy with corresponding periumbilical, suprapubic, and left lateral quadrant port placements. During subsequent evaluation, the appendix was noted to be normal in nature without signs of inflammation. The appendix was taken in typical fashion, and the remainder of the bowel was evaluated for possible underlying pathology. Upon evaluation near the ligament of Treitz, 2 loops of small bowel were noted near the fourth portion of the duodenum entering what appeared to be a defect left lateral to the duodenum with one loop of bowel exiting and one entering. 40 cm of injected and dilated small bowel was manually reduced from the left-sided paraduodenal hernia defect. A fourth trocar was placed in the right supraumbilical region to provide greater retraction. However, due to poor initial port placement as a result of planned laparoscopic appendectomy, inability to progress, and lack of experience with PDH disease process, the repair of the paraduodenal hernia defect was not performed during that procedure.

The patient was discharged 24 hours later with plans for outpatient evaluation and repair; however, she returned approximately 72 hours later with similar complaints of abdominal bloating, nausea emesis, and persistent right lower quadrant abdominal tenderness. Due to the prior established diagnosis, the patient underwent a robotic left-sided paraduodenal hernia repair using the prior supraumbilical, right lower quadrant, and suprapubic ports with two additional ports placed on the left side for 4-arm docking. The 4 cm defect was identified after elevation of the transverse mesocolon, and multiple loops of small bowel were reduced out of the paraduodenal hernia with repositioning in reverse Trendelenburg position. The defect was closed with interrupted silk suture between the peritoneum alongside the inferior mesenteric vein and the wall of the duodenum as it exited the ligament of Treitz. Follow-up evaluation in the year since operation has showed complete resolution of all symptomatology.

It should be noted that the choice to not complete the repair of the PDH at the index operation placed the patient at increased risk for reoccurrence and possible strangulation of bowel. Simple reduction without repair of internal hernia defects is not recommended. A preferred approach if unable to complete the repair laparoscopically secondarily due to port positions is to place more ports. Another approach is to convert to an exploratory laparotomy. The repair of PDH from a midline incision does not differ from the principles of laparoscopic repair with obliteration of the defect with a primary repair remaining the goal.

## 4. Discussion

Due to relative rarity of paraduodenal hernias, the identification and repair of PDH is less common in general surgical practice. They have a mean age of diagnosis of 38.5 years [2], with approximately 75% of all occurring on the left-hand side and a 3 : 1 male gender distribution [9]. Typically, patients present with recurrent undiagnosed abdominal pain, postprandial pain, and symptomatology to suggest bowel obstruction or CT evidence of bowel obstruction without prior evidence of surgical intervention. CT imaging of the abdomen and pelvis with IV and by mouth contrast is the preferred modality; however, it only has a 43% [13] predicted value of identifying a paraduodenal hernia. In our small case series, successful identification of PDH by radiologist interpretation occurred in one of the three patients. Subsequent diagnosis was completed by surgical staff members in the setting of undiagnosed reoccurring and relapsing abdominal pain and bowel obstructive-type symptoms.

**Figure 4:**
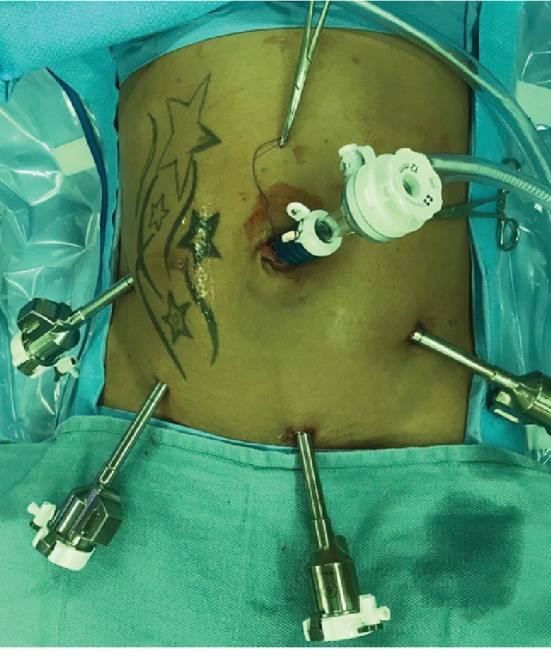
Robotic port setup for operative repair of left paraduodenal hernia.

In our case series, one left PDH was repaired robotically and two were repaired laparoscopically. The single robotic case was completed with the da Vinci Si surgical system with a 12 mm periumbilical trocar and four total 5 mm trocars (Figure 4) placed at the right midabdomen, right lower quadrant, left lower quadrant, and suprapubic. The case began with running the small bowel from the cecum to the ligament of Treitz. There were multiple pauses during the operation to undock the robot and facilitate patient positioning in Trendelenburg, reverse Trendelenburg, right or left side up. Upon arriving at the ligament of Treitz, the small bowel was manually reduced in typical fashion and the defect was repaired with a single layer of absorbable Vloc suture in running fashion to approximate the jejunum to the transverse mesocolon. During the surgery, the location of the suprapubic 5 mm trocar seen in Figure 4 was unusable during manipulation near the ligament of Treitz due to robotic arm limitations and later felt to be unnecessary in future procedures.

**Figure 5:**
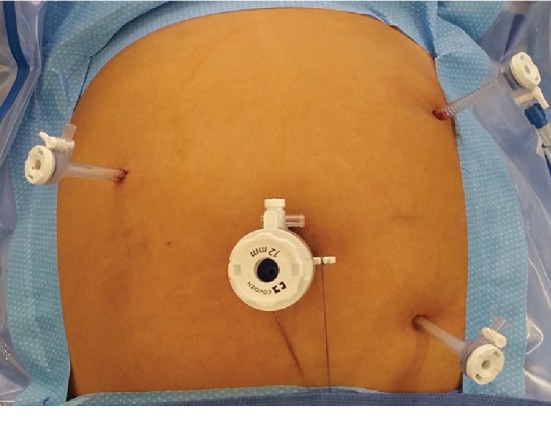
Laparoscopic port placement for operative repair of left paraduodenal hernia.

A total of 4 trocars were placed during laparoscopic repair of left paraduodenal hernias: one 12 mm periumbilical and three 5 mm (Figure 5). The 5 mm trocars were placed in the right upper quadrant, left upper quadrant, and left lower quadrant. The camera was placed in the 12 mm port. To begin the procedure, the left upper quadrant and left lower quadrant ports were used to run the bowel from the cecum towards the ligament of Treitz. Near the ligament, the working ports were transitioned to the right upper quadrant and periumbilical ports. The camera was placed in the left lower quadrant port, and the left upper quadrant port was used to elevate the transverse colon and provide better visualization to aid in the identification of the ligament of Treitz. The abovementioned port placement is ideal to maximize mobility while minimizing the amount of port access. This arrangement freely allows the operator to use the Endo Stitch in the right hand and duckbill in the left hand via the right upper quadrant port to complete the repair. The assistant port in the left upper quadrant provides essential exposure to the area of repair. Laparoscopic repair of PDH is more conducive versus robotic repair because of efficiency of patient repositioning. Repositioning in steep Trendelenburg, reverse Trendelenburg, and right or left side up is better achieved without having to redock the robot after each change in patient body position. An Endo Stitch running repair was performed on the defect in standard fashion using absorbable suture with bites incorporating the first portion of the jejunum, transverse colon, and mesocolon. Subsequent running of this suture obliterated the defect with minimal difficulty.

## 5. Conclusion

Internal hernias are an uncommon cause of small bowel obstruction. Paraduodenal hernias represent a subset of internal hernias and are found on autopsy in 0.2-0.9% [4] of the population. Although rare, the adverse outcome of an unsuspected or unidentified paraduodenal hernia can result in ischemia, strangulation, and obstruction, with mortality between 20 and 50% due to delayed management [2]. CT scan with IV and by mouth contrast of the abdomen and pelvis is the recommended modality for identification of paired duodenal hernias; however, only 43% of cases are correctly identified on radiologist interpretation [13]. Patients with relapsing and remitting abdominal discomfort, postprandial pain, and history suggestive of obstructive-type pathology are the identifying factors in our case series. Surgical staff members should always have a high index of suspicion for PDH in patients with evocative presentations. It necessitates a thorough review of CT imaging for displacement of bowel, atypical anatomy, and consideration for diagnostic laparoscopy. Laparoscopic repair of left-sided paraduodenal hernias may be the preferred method over robotic repair based upon available instrumentation. During surgical evaluation of an internal hernia, patient repositioning allows for efficient running of the bowel from the cecum to the ligament of Treitz. Current technology using the da Vinci Si surgical system requires detachment and redocking multiple times to complete this task. The actual repair of the defect after reduction of the PDH may be easier via robot due to additional degrees of freedom and use of Vloc suture to avoid intracorporeal knot tying. However, a skilled laparoscopic surgeon can perform the same repair with an Endo Stitch within a similar amount of time. The crucial element to an efficient paraduodenal hernia repair is optimal port placement. In our case series of two laparoscopic repairs, the ideal port placement is a 4-port placement as described above. This allows for full evaluation of the small bowel, elevation of the transverse colon along with repair of the mesenteric defect. It is our hope that this small case series will aid in future surgeons' ability to identify and repair paraduodenal hernias with little difficulty.
